# Mineral-Associated Soil Carbon is Resistant to Drought but Sensitive to Legumes and Microbial Biomass in an Australian Grassland

**DOI:** 10.1007/s10021-017-0152-x

**Published:** 2017-04-25

**Authors:** Alberto Canarini, Pierre Mariotte, Lachlan Ingram, Andrew Merchant, Feike A. Dijkstra

**Affiliations:** 1 Centre for Carbon, Water and Food, School of Life and Environmental Sciences, The University of Sydney, 380 Werombi Road, Camden, NSW 2570 Australia; 2 Department of Microbiology and Ecosystem Science, University of Vienna, Althanstr. 14, 1090 Vienna, Austria; 3 Laboratory of Ecological Systems (ECOS), School of Architecture, Civil and Environmental Engineering (ENAC), Ecole Polytechnique Fédérale de Lausanne (EPFL), Station 2, 1015 Lausanne, Switzerland; 4 Swiss Federal Institute for Forest, Snow and Landscape Research (WSL), Site Lausanne, Case postale 96, 1015 Lausanne, Switzerland

**Keywords:** carbon storage, carbon inputs, microbial biomass carbon, organo-mineral carbon, plant functional groups, plant–soil interactions, rainout shelter

## Abstract

Drought is predicted to increase in many areas of the world with consequences for soil carbon (C) dynamics. Plant litter, root exudates and microbial biomass can be used as C substrates to form organo-mineral complexes. Drought effects on plants and microbes could potentially compromise these relative stable soil C pools, by reducing plant C inputs and/or microbial activity. We conducted a 2-year drought experiment using rainout shelters in a semi-natural grassland. We measured aboveground biomass and C and nitrogen (N) in particulate organic matter (Pom), the organo-mineral fraction (Omin), and microbial biomass within the first 15 cm of soil. Aboveground plant biomass was reduced by 50% under drought in both years, but only the dominant C4 grasses were significantly affected. Soil C pools were not affected by drought, but were significantly higher in the relatively wet second year compared to the first year. Omin-C was positively related to microbial C during the first year, and positively related to clay and silt content in the second year. Increases in Omin-C in the second year were explained by increases in legume biomass and its effect on Pom-N and microbial biomass N (MBN) through structural equation modeling. In conclusion, soil C pools were unaffected by the drought treatment. Drought resistant legumes enhanced formation of organo-mineral complexes through increasing Pom-N and MBN. Our findings also indicate the importance of microbes for the formation of Omin-C as long as soil minerals have not reached their maximum capacity to bind with C (that is, saturation).

**Electronic supplementary material:**

The online version of this article (doi:10.1007/s10021-017-0152-x) contains supplementary material, which is available to authorized users.

## Introduction

Grassland ecosystems represent between 30 and 40% of the global land surface area, storing organic C in amounts comparable to forest ecosystems (White and others 2000). Environmental stresses can cause loss of C from terrestrial ecosystems, thereby increasing the atmospheric CO_2_ concentration and global warming potential. Foremost, water stress (that is, drought) can turn grassland ecosystems into C sources (Zhang and others 2010, 2011; Hoover and Rogers 2016), specifically by reducing net primary production (NPP; that is, C input to soil). Soil organic matter (SOM) decomposition (that is, C outputs from soil) is often maintained (Hoover and Rogers 2016), although, the response of SOM decomposition to drought will depend on drought intensity and timing of rewetting periods (Bloor and Bardgett 2012). Because SOM is made of C pools with different inherent levels of turnover and stability (Six and others 2002), understanding the C dynamics of different pools in response to environmental stress is critical to assess impacts of grassland ecosystems on CO_2_ release to the atmosphere. Therefore, understanding drought effects on C pools is particularly important for predicting climate change feedbacks in grassland ecosystems and possible legacy effects of post-drought periods.

Particulate organic matter (composed of plant litter and organic amendments in agricultural systems) is considered to have faster turnover times compared to organic matter bound to the soil mineral fraction (organo-mineral fraction) and that is considered more resistant to microbial mineralization (Cotrufo and others 2013; Feng and others 2014; Ahrens and others 2015). This mineral-associated pool of C is primarily determined by soil mineralogy as well as by plant and microbial inputs (Kögel-Knabner and others 2008; Cotrufo and others 2013). Organo-mineral C (Omin-C) is formed upon binding of organic matter (OM) to clay and silt (Mikutta and Kaiser 2011). Recently, it was suggested that the main source of C binding to the mineral fraction comes from plant-derived labile compounds (Cotrufo and others 2015). These labile compounds (either leachates from particulate organic matter decomposition or root exudates) can directly bind to the mineral fraction or can be incorporated into microbial biomass before it is bound to the mineral fraction (Castellano and others 2015). Indeed, microbial biomass was identified as a primary constituent of the organic matter in organo-mineral complexes (Solomon and others 2012; Kögel-Knabner 2017). The microbial pathway is supported by our previous experiment where we showed that plant-derived C in microbial biomass had a positive relationship with plant-derived C in the organo-mineral complexes (Canarini and Dijkstra 2015). Moreover, it has been shown that the efficiency at which microbes utilize plant compounds is positively correlated to the amount of soil C formed (Bradford and others 2013).

In a recent framework, Cotrufo and others (2013) suggested that plant inputs of low C/N ratio are preferentially utilized by microbes and ultimately incorporated into organo-mineral complexes (the *Microbial Efficiency*-*Matrix Stabilization* (MEMS) framework).Variation in the C/N ratio of plant community inputs associated with different plant functional groups (Aerts and Chapin III 1999; Kerkhoff and others 2006; Hobbie 2015; Canarini and others 2016a) could therefore result in differences in both the microbial activity (Enríquez and others 1993) and soil C storage (De Deyn and others 2008). Indeed, the presence of specific plant functional traits, such as biological N-fixation (that is, legumes), is recognized as an important driver of C accumulation in grasslands (Fornara and Tilman 2008; De Deyn and others 2009, 2011) and cropland soils (Kallenbach and others 2015; Frasier and others 2016). At the same time, plants have the ability to cause desorption of organic material bound to minerals through release of organic acids (Keiluweit and others 2015). All this highlights the control of plants and the plant community over soil C pools.

Drought effects on plants, microbes and their interactions could have indirect negative outcomes for organo-mineral complexes. However, information about drought effects on plant–microbe control over organo-mineral C is limited. Water availability can greatly impact NPP and shape the plant community composition (Yang and others 2011; Hoover and others 2015), depending on the stress intensity. For example, dominant species are often more sensitive to drought than subordinate species in grassland communities and decrease in biomass during water stress (Mariotte and others 2013). Because the plant community is an important player controlling soil processes (Díaz and Cabido 2001; Fornara and Tilman 2008; De Deyn and others 2009), drought-induced changes in plant community composition and structure could have significant impacts on C storage. Drought can also reduce the flux of C from root exudates (Kuzyakov and Gavrichkova 2010), which greatly contributes to microbial activity in soil (Shahzad and others 2015). At the same time, water stress limits the diffusion of substrates and slows down biological processes, directly reducing soil microbial activity (Schimel and others 2007), although rewetting periods following drought can enhance decomposition, thereby offsetting the reduction in decomposition during the drought period (Borken and Matzner 2009).

The aim of this study was to investigate drought effects on two soil C pools of different inherent turnover and stability (particulate organic C, or Pom-C, and Omin-C) and whether the C content of these pools was related to the abundance of specific plant functional groups, microbial biomass and soil nutrients. A drought manipulation (that is, reduced precipitation) experiment was undertaken in an Australian grassland using rainout shelters and compared to an ambient precipitation control. Previously, we found that one year of drought manipulation had no effect on Omin-C, but that this pool was positively related to fungi in the top 5 cm of the soil and to gram-negative bacteria deeper in the soil profile (5–15 cm; Canarini and others 2016b). Here, we assess drought effects on Omin-C through changes in plant community structure and microbial biomass spanning 2 years of water manipulation. We hypothesized that:

(i) drought would reduce plant biomass and C inputs, thereby decreasing particulate organic C (Pom-C), microbial biomass C and to a lesser extent Omin-C;
(ii) microbial biomass C would relate positively to Omin-C because microbial biomass is the primary substrate for the formation of this pool;
(iii) drought effects on Omin-C formation would be mediated by plant functional group responses to drought.

## Materials and Methods

### Site Description and Experimental Design

This study was conducted in a semi-natural grassland at John Bruce Pye Farm on the campus of The University of Sydney, NSW, Australia (33°55′51″S, 150°39′38″E). The site is at 81 m above sea level with a maximum temperature of 29.8°C in January and a minimum temperature of 4.1°C in July (20-year average, Australian Bureau of Meteorology). The mean annual precipitation recorded over the last 20 years is 692 mm, ranging between 414 and 1041 mm (Bureau of Meteorology 2016). Precipitation, in the form of rain or hail, is slightly higher in February compared to the rest of the year. Pastures are commonly fertilized every year depending on soil fertility and usually applied as urea and superphosphate, and they are regularly grazed by cattle though these pastures had not had fertilizer applied for more than 5 years. The vegetation (*see* full list in Table S1) is dominated by C4 grasses (*Paspalum dilatatum*, *Paspalum distichum*, *Cyperus brevifolius*, *Setaria incrassata*) and a C3 grass (*Microlaena stipoides*). Soil is a clay loam and is classified as a red-brown Chromosol according to the Australian Soil Classification (Isbell 2002). Average values for soil characteristics in the top 15 cm are: clay 35%, sand 34%, silt 31%, organic C% 3.2, N% 0.23, P% 0.015, pH 5.7 (H_2_O). Cattle were excluded during the experiment, but grazing was simulated by clipping plant biomass in plots and mowing the area around plots twice a year in May and January.

In January 2014, we selected an area (23 × 17 m) of uniform grass density and established 16 plots (2 × 2 m) in a block design. The area is on a 10% slope with a northerly aspect, and we accounted for the slope by placing 4 blocks parallel to the slope with each block containing 4 plots. Distance between plots was 3 m within each block and 5 m between blocks. In each block, two of the four plots were randomly assigned to the drought treatment (50% reduction in ambient precipitation), whereas the other two plots received ambient precipitation (control plots). To simulate drought, rainout shelters (2 m width × 2 m length × 1 m height) were constructed following the design of Yahdjian and Sala (2002). Clear acrylic U-shaped gutters, transmitting almost all of the visible light, were used to intercept half of the ambient precipitation. Intercepted precipitation was diverted away from the plots with separate gutters. A plastic barrier was buried 40 cm deep into the ground at the side of each plot facing the slope to prevent runoff and lateral flow going into the plot. To control for shelter effects on UV light interception, shading and temperature, the same shelters were installed in control plots, but mounted upside down to let all precipitation through. In addition, a fertilizer treatment was included, where fertilizer was added either in mineral form (40 kg ha^−1^ y^−1^ N, 10 kg ha^−1^ y^−1^ P, and 16 kg ha^−1^ y^−1^ K) or as compost (6 t ha^−1^ y^−1^ of compost). The fertilizer treatment was added in interaction with the drought treatment, but as there were no significant fertilizer effects on the parameters measured in this study, they will not be discussed here (but *see* Canarini and others 2016b).

Sampling only occurred in the central 1 m square subplot, whereas the surrounding area (50 cm wide on all four sides) served as buffer zone. Temperature (S-TMB-M0XX) and moisture probes (S-SMx-M005) were installed at 5 cm soil depth in each subplot of the first and last block on the 15th of April 2014, and temperature and moisture were recorded every 15 min on a data logger (HOBO U30 station, Onset Computer Corporation, Bourne, MA, USA). Other meteorological data were obtained from the Badgerys Creek meteorological station (Bureau of Meteorology 2016) located about 7 km from the experimental site.

### Soil Sampling and Analysis

Soil samples were collected in January 2015 and January 2016 in each subplot in the first 15 cm of the soil profile. Three soil cores (2.5 cm in diameter) were collected and pooled together before analysis, for a total of 32 samples (16 plots × 2 years). Soil samples were sieved (2 mm) and homogenized on site, brought to the laboratory and kept at 4°C until analyzed on the following day.

All soil samples were analyzed for gravimetric water content, microbial biomass carbon (MBC), nitrogen (MBN) and phosphorus (MBP), dissolved organic C (DOC), dissolved organic nitrogen (DON), nitrate (NO_3_^−^), ammonium (NH_4_^+^) and available P. Gravimetric water content was measured by drying the soil at 65°C for 72 h. The chloroform fumigation-extraction technique (Vance and others 1987) was used to determine MBC, MBN and MBP. For MBC and MBN, 5 g of soil was weighed in two different containers. The first set of containers received 40 ml of 0.05 M K_2_SO_4_ solution and was shaken for 1 h. Samples were centrifuged at 4000 rpm for 5 min, before being filtered through Whatman #42. The other set of samples was fumigated in a desiccator with chloroform for 72 h and then extracted following the same procedure as the non-fumigated samples. Fumigated and non-fumigated K_2_SO_4_ extracts were acidified with 0.2 ml 1 M phosphoric acid and analyzed for total organic C and N using a TOC-N analyzer (Shimadzu TOC-V csh, TNM-1, Kyoto, Japan). The difference between fumigated and non-fumigated samples was divided by 0.45 (Vance and others 1987) and considered as the MBC, while a factor of 0.54 (Brookes and others 1985) was used to calculate MBN. Total organic C and N in the non-fumigated samples were considered as DOC and total dissolved nitrogen (TDN), respectively. NO_3_^−^ and NH_4_^+^ were measured on a Flow Injection Analyzer (FIA automated ion analyzer, Lachat Instruments, Loveland, CO, USA), and DON was obtained by subtracting their combined values from TDN. Phosphorus was extracted from 3 g of soil with 20 ml 0.03 M NH_4_F—0.025 N HCl and fumigation lasted 24 h. The P concentration was measured colorimetrically using the ammonium molybdate–stannous chloride reagent (Olsen and Sommers 1982). The difference between fumigated and non-fumigated samples was divided by 0.4 (Hedley and Stewart 1982) and considered as MBP, whereas P in the non-fumigated samples were considered as a measure of available P. Results of these analyses for soils sampled in January 2015 were previously reported in Canarini and others (2016b) with the exception of MBC, MBN and MBP.

### Soil C Fractionation

Dry soil samples were separated into a soil particulate organic matter fraction (Pom-C and Pom-N) and a mineral fraction (Omin-C and Min-N) by dispersion in sodium hexametaphosphate, followed by wet sieving using a 53 μm sieve (Canarini and Dijkstra 2015). The mineral fraction includes the silt and clay fraction, whereas the particulate organic matter includes sand. Although methods for determining meaningful SOC fractions all possess shortcomings (Olk and Gregorich 2006; von Lützow and others 2007), mineral-associated C determined with size fractionation methods is expected to have slower turnover times and greater long-term sink capacity than Pom-C (Schlesinger and Lichter 2001). This expectation was supported by higher δ^13^C values (Collins and others 2000; Bradford and others 2008) indicating that mineral-associated C has been processed to a greater extent. Further, C in the Pom fraction is largely plant-derived, whereas that in the mineral fraction is largely derived from microbial material (Grandy and Robertson 2007; von Lützow and others 2007). After fractionation, soil samples were dried, ground and analyzed for total C and N on an isotope ratio mass spectrometer (Delta V Advantage with a Conflo IV interface, ThermoFisher Scientific, Bremen, Germany). Samples from 2015 were also run for total C and N using a CHN analyzer (LECO TruSpec CHN, USA), and results were in good agreement with the results from the isotope ratio mass spectrometer (*P* < 0.001, *r* = 0.98).

### Plant Harvesting

Plots were clipped twice a year during peak biomass in January and after re-growth in May to approximately 4 cm above the soil surface from a 50 × 50 cm quadrat within the central subplot. The remainder of the plot was mowed with a lawnmower, and all aboveground biomass was removed from the plots. Plant biomass harvested in January 2015 and 2016 was sorted by species, dried at 65°C during 72 h and weighed. Plant biomass of each species was then grouped into four different functional groups: C4 and C3 grasses, forbs and legumes. Plant biomass harvested in May was not sorted by species because the percentage of undetermined species was large due to low growth and flowers presence, but otherwise processed in the same way.

### Data Analysis

A two-way repeated measures analysis of variance (ANOVA) was used to test for main and interactive effects of drought and time on all soil parameters and on total aboveground biomass and for each functional group (C4, C3, forbs and legumes), with block as error term. ANOVAs were performed in JMP v. 8.0.1 (SAS Institute, Cary, NC, USA). We compared Euclidean distances of plant community and soil parameters using permutational multivariate analysis of variance (PerMANOVA) to determine effects of drought and time on the structure of plant community and soil properties. Linear regressions and correlations were carried out to examine relationships between different soil parameters and total aboveground biomass and plant functional group biomass. Linear regression was applied when literature suggested causality (for example, aboveground biomass or MBC *vs* Omin-C), whereas correlations were used when causal relationships were more uncertain. Statistical significance of linear regressions was obtained from ANOVAs with the *R* ^*2*^ indicating the goodness-of-fit. Statistical significance for linear correlation was obtained after calculating Pearson’s correlation coefficient (*r*).

Significant results were linked using a priori knowledge in a path analysis model to determine the linkages between temporal changes (between 2015 and 2016) in plant community structure and soil properties with temporal changes in Omin-C. Path analysis represents a special class of structural equation modeling (SEM) containing only observed variables, where SEMs are probabilistic models that can include multiple predictors and response variables in a single causal network. A full model was built including all hypotheses (for full list of a priori hypothetical pathways see Supplementary information, Figure S1). We tested two separate models, one model containing MBC and Pom-C, the other where MBC and Pom-C were replaced with MBN and Pom-N. The full models were simplified by step-wise exclusion of nonsignificant variables (either by weights or covariance) as estimated by AIC (Akaike information criterion), until a minimum adequate model was reached (Milcu and others 2013). The adequacy of the models was determined by nonsignificant Chi-square tests (*χ* ^2^, *P* > 0.05), low root-mean-square error of approximation index (RMSEA < 0.1), Tucker-Lewis Index (TLI ≥ 0.90) and high comparative fit index (CFI ≥ 0.90) (Grace and others 2010). Linear regressions, correlations, PerMANOVA and SEM were performed in the R language environment version 3.2.1 (R Core Team 2013).

## Results

### Soil Biotic and Abiotic Variables

**Figure 1:**
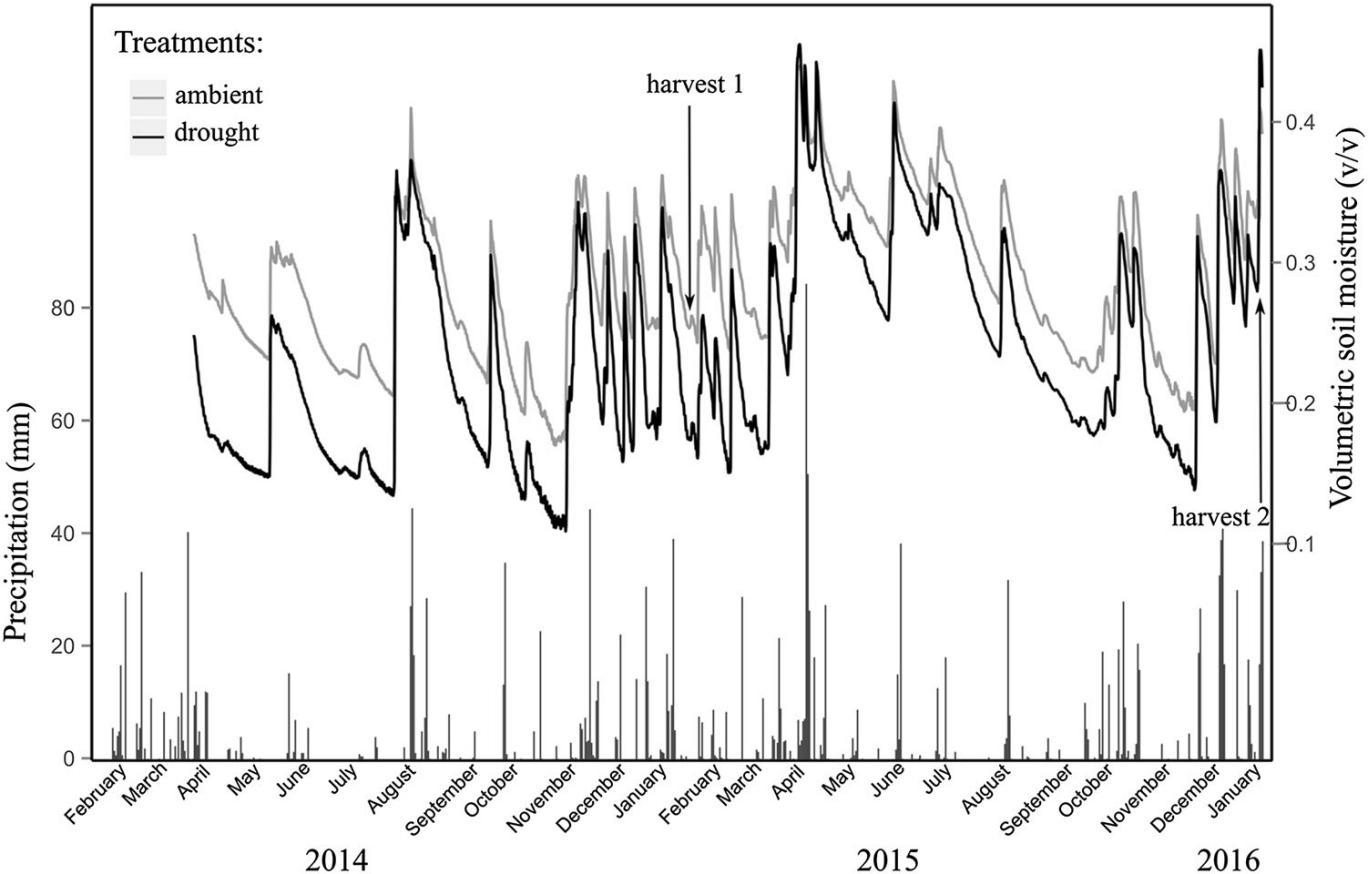
Volumetric soil moisture content (v/v) and precipitation (mm) from February 2014 to January 2016. Soil moisture content is shown for ambient and reduced precipitation treatments and averaged from the first and last block (*n* = 4). Arrows indicate time of sampling for 2015 (harvest 1) and 2016 (harvest 2).

**Figure 2:**
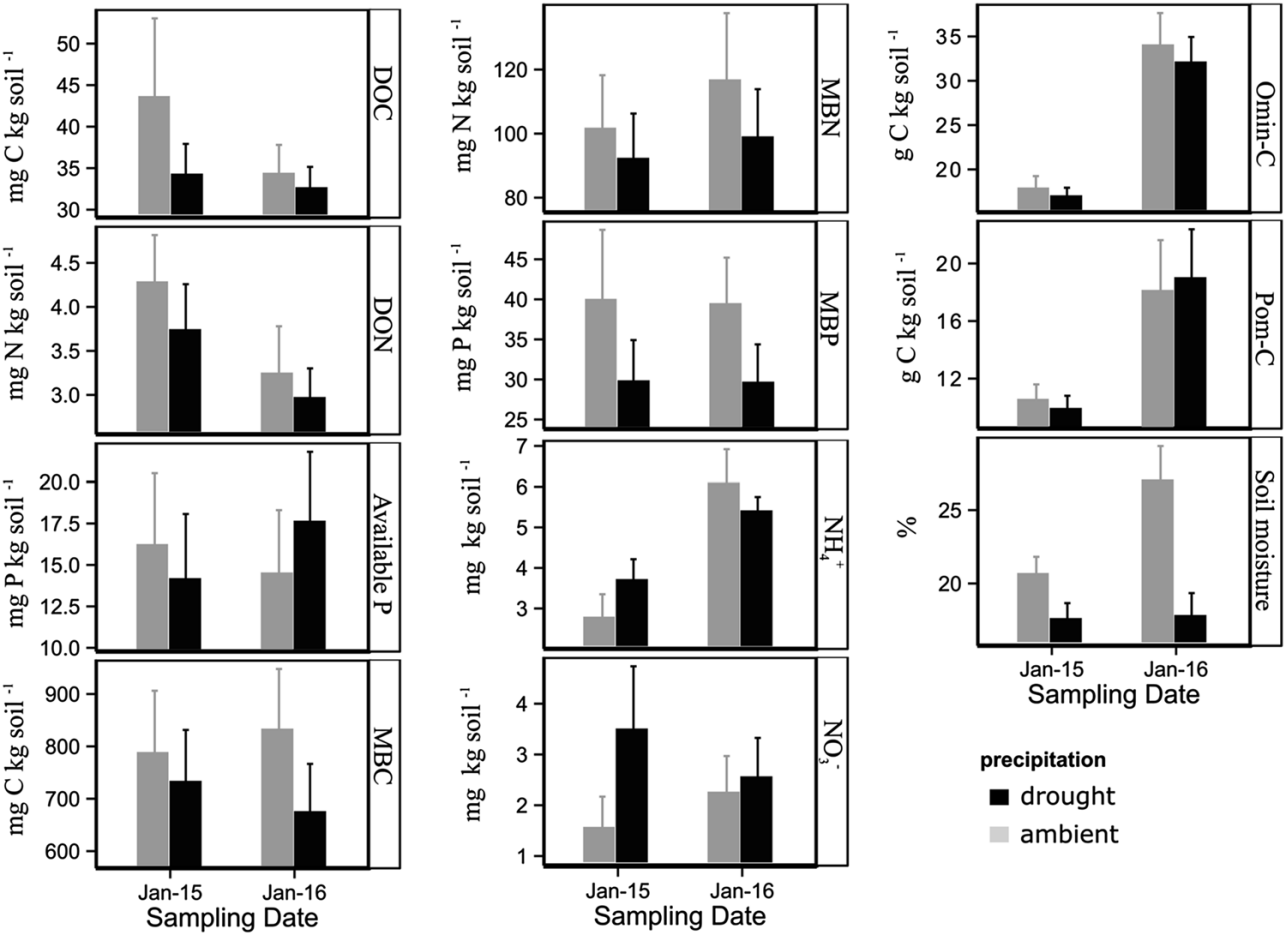
Mean values of dissolved organic C (DOC) and N (DON), available P, microbial biomass C (MBC), N (MBN), and P (MBP), soil extractable NH_4_^+^ and NO_3_^−^, organo-mineral C (Omin-C), particulate organic C (Pom-C) and soil moisture, for both the drought and ambient precipitation treatments at the two sampling dates (January 2015 and 2016). Error bars represent standard error of the means.

Overall, the drought treatment successfully reduced soil moisture content (Figure 1). In 2014, the reduction in soil moisture (23% across all probe measurements) was greater (compared to ambient control) than in 2015 (14%). Correspondingly, annual rainfall in 2015 was 820 mm compared to 693 mm in 2014, leading to more periods in which ambient and reduced precipitation treatments had similar soil moisture content due to periods of intense precipitation. Nevertheless, the effect of the drought treatment resulted in a significant reduction in the gravimetric soil moisture measured in soil samples (Table 1; Figure 2).

**Table 1:** ANOVA and PerMANOVA-P Values of Soil Parameters for Main and Interactive Effects of Drought and Time

Factor	Soil parameter											PerMANOVA
	Soil moisture	MBC	MBN	MBP	Min-C	Pom-C	Pom-N	NH_4_^+^	NO_3_^−^	P	DON	
Drought	**0.001**	n.s.	n.s.	**0.010**	n.s.	n.s.	n.s.	n.s.	n.s.	n.s.	n.s.	**0.031**
Time	**0.006**	n.s.	**<0.001**	n.s.	**<0.001**	**<0.001**	0.09	**<0.001**	n.s.	n.s.	**0.016**	n.s
Drought × time	**0.015**	**0.020**	n.s.	n.s.	n.s.	n.s.	n.s.	**0.024**	n.s.	n.s.	n.s.	n.s.

*P* values are reported in bold when *P* < 0.05.

**Figure 3:**
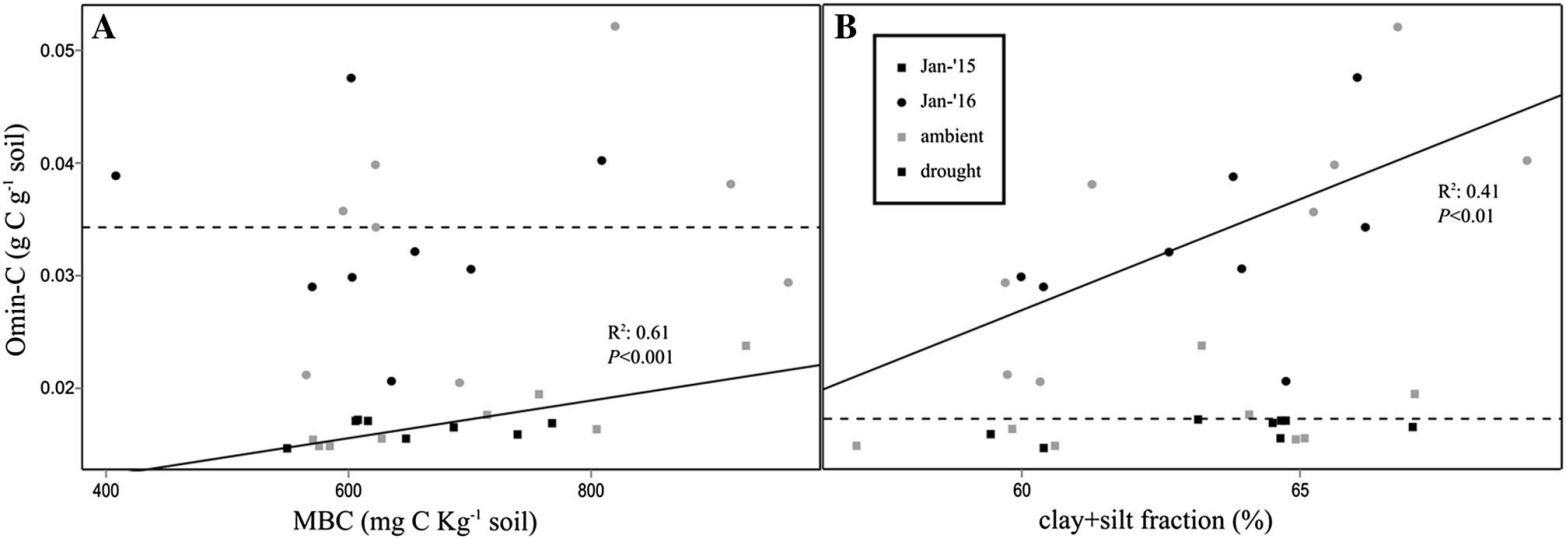
Relationship between organo-mineral C (Omin-C) and **A** microbial biomass C (MBC) and **B** the clay/silt fraction in the soil. The *horizontal dotted lines* indicate the mean of values corresponding to January 2016 (**A**) and January 2015 (**B**), respectively, as no significant linear regression was found. The *continuous lines* represent significant linear regressions in **A** for 2015 and **B** 2016 and *R* ^*2*^ and ANOVA *P* values are reported next to each line. *Square symbols* represent samples from January 2015 whereas *circles* from January 2016; *gray symbols* represent ambient treatment and black drought treatment.

The drought treatment significantly decreased MBP (Table 1; Figure 2) but there were no significant effects for all other soil parameters. However, a significant positive relationship was found between soil moisture and MBC when linear regression was applied (*R* ^*2*^ = 0.43, *P* < 0.001). Time was almost always significant, causing an increase in MBN, NH_4_^+^, Omin-C and Pom-C, and a decrease in DON in January 2016 compared to January 2015. MBC was positively related with Omin-C in January 2015, but this relationship was lost in January 2016 (Figure 3A). When Omin-C content was regressed against the clay/silt fraction (the mineral fraction associated with Omin-C and expressed as a percentage of the total soil mass), a significant relationship was found in 2016, but not in 2015 (Figure 3B). Soil properties presented in Table 1 were also analyzed with PerMANOVA, and were significantly affected by drought (*P* = 0.031), but not by main and interactive effects of time.

### Aboveground Plant Biomass

**Figure 4:**
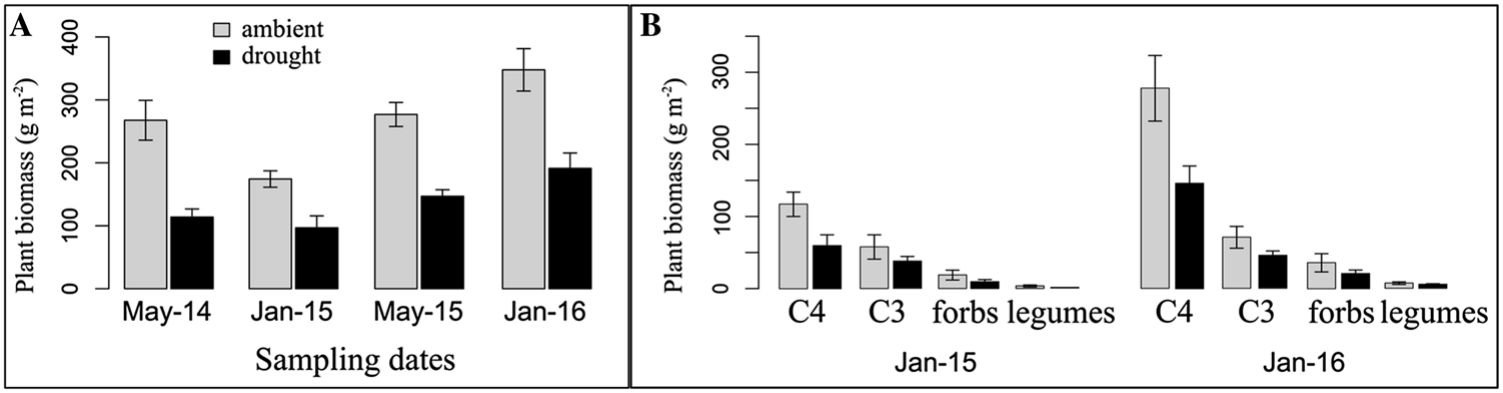
Mean values (g m^−2^) for total aboveground biomass (**A**) and aboveground biomass of each functional group (**B**). Total aboveground biomass values are reported for all sampling dates and treatments. Dates are: May and January 2015 and 2016 (reported as May or Jan, -15 or -16). Treatments are: ambient and drought. The biomass of plant functional groups is reported for the two sampling dates where species were identified (January 2015 and 2016) and for both the drought and ambient precipitation treatments. *Error bars* represent standard error of the means.

Aboveground plant biomass decreased with drought in both years (*P* < 0.01 in 2015 and *P* < 0.01 in 2016), although aboveground plant biomass doubled in January 2016 compared to January 2015 (Figure 4A). Most of the drought-induced reduction in aboveground biomass was caused by a decrease in the biomass of C4 grasses (*P* = 0.02), whereas the biomass of C3 grasses, forbs and legumes was not affected (Figure 4B). When the whole plant community composition was analyzed with PerMANOVA, we did not observe a significant change due to drought, while plant community changes were mostly due to time effects (*P* = 0.002).

### Plant–Soil Interactions

**Figure 5:**
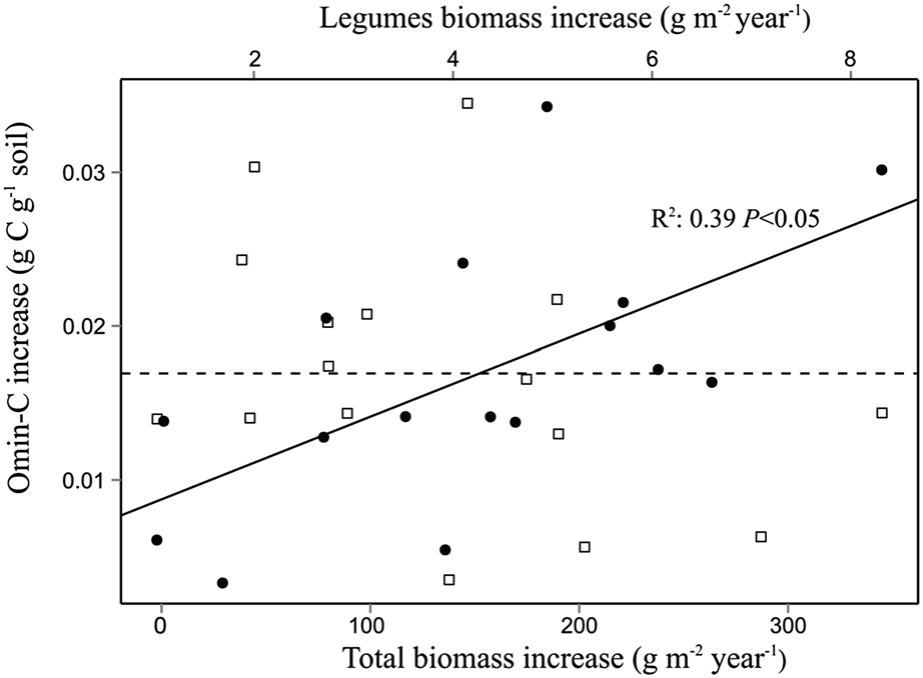
Relationship between the increase in organo-mineral C (Omin-C) between January 2015 and January 2016 (data from 2015 were subtracted from 2016) and the increase in aboveground biomass of legumes (*continuous line*) and the total aboveground biomass (*dotted line*). The *dotted line* corresponds to the mean, as no significant linear regression was found. The *continuous line* represents significant linear regression, where *R* ^*2*^ and ANOVA *P* values are reported.

When Omin-C was correlated with plant functional group biomass, we observed significant correlations with legumes, forbs and total aboveground biomass, although the strength of the relationship was in the order: legumes > forbs > total biomass (legumes: *R* ^*2*^ = 0.38, *P* < 0.001; forbs: *R* ^*2*^ = 0.28, *P* < 0.002; total biomass: *R* ^*2*^ = 0.17, *P* = 0.02). We found no significant relationship between the increase (2016 compared to 2015 measurements) in soil Omin-C and the increase in total aboveground plant biomass (Figure 5). However, we found a significant positive relationship between the increase in Omin-C and the increase in aboveground biomass of legumes (Figure 5). We observed a positive linear correlation between the biomass of C4 and legumes and soil moisture (*r* = 0.68 and *r* = 0.45, respectively; *P* < 0.05), but not for the other functional groups. An increase in legume biomass was positively correlated with Pom-N and MBN (*r* = 0.43 and *r* = 0.35, respectively; *P* < 0.05).

**Figure 6:**
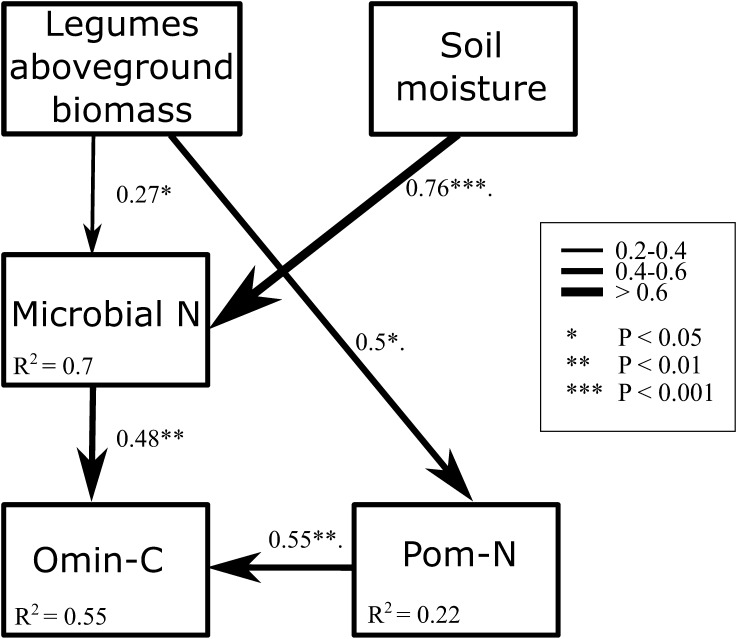
Structural equation model for the effect of temporal changes in legume biomass and soil moisture on changes in particulate organic N (Pom-N), microbial biomass N (Microbial N) and organo-mineral C (Omin-C) between 2015 and 2016. *Boxes* represent changes in pools between 2015 and 2016. *Arrows* with different width represent different standardized effect sizes as shown in the legend. Significant values are indicated by *(*P* < 0.05), **(*P* < 0.01) and ***(*P* < 0.001). *R* ^2^ values are indicated for the dependent variables. Our overall model fit was satisfactory (*χ* ^2^ = 5.6, *P* = 0.23; TLI = 0.9; CFI = 0.95; RMSEA = 0.157, confidence intervals: 0 and 0.434, *P* = 0.461).

We investigated interactions between plants and soil microbial biomass and their impacts on soil C pools through SEM, to explain the increase found in Omin-C between the two years of treatment. We were unable to find a significant model using MBC and Pom-C. However, after step-wise exclusion of nonsignificant variables (changes in total plant biomass and available soil N) a significant model was found (Figure 6). Changes in legume biomass between 2015 and 2016 were positively related to changes in Pom-N and MBN, which in return were positively related to changes in Omin-C. Furthermore, changes in MBN were also positively affected by soil moisture changes. The fitting parameters for this SEM were excellent (Figure 6), and the model explained 55% of the variance in Omin-C increase between 2015 and 2016.

## Discussion

Direct effects of water limitation to plant and microbes may reduce C inputs to soil and slow down organic matter processing (Borken and others 2006; Cook and Orchard 2008). However, previous field studies showed that drought in grasslands decreased NPP although soil organic matter decomposition was less affected (Hoover and Rogers 2016; Lei and others 2016). This could be due to drying and rewetting cycles that enhance soil organic matter decomposition compensating for the direct inhibitory effects of drought on microbial activity (Borken and Matzner, 2009), but also from increased plant C allocation belowground (Sanaullah and others 2012). In our study, two years of reduced precipitation caused a significant reduction in total plant biomass (halved in both years, Figure 4A). This decrease was mainly due to a decrease in the biomass of C4 grasses (Figure 4B), which represents the dominant group of species. However, both Pom-C and Omin-C pools showed no differences compared to the control, contrary to our first hypothesis. This result suggests a likely change in shoot to root ratio, with more C allocated belowground, which would explain the reduction in aboveground biomass despite the persistence of Pom-C under the drought treatment. Indeed, belowground biomass might represent a better indicator of C inputs (Fornara and Tilman 2008), especially because belowground biomass typically increases more than aboveground biomass in grazed systems (Schuman and others 1999; Chen and others 2015). In addition, because aboveground biomass was removed from the plots twice a year, aboveground plant litter inputs were strongly reduced, making inputs through rhizodeposition (root litter or root exudates) potentially more important. Unfortunately, we did not collect belowground biomass nor measured rhizodeposition and thus can only speculate about the C allocation to root biomass.

Microbial biomass can act as an intermediate by decomposing plant inputs (that is, litter or root exudates) and supplying C to organo-mineral complexes (Omin-C; Cotrufo and others 2013). Therefore, we hypothesized that microbial biomass would relate to Omin-C (second hypothesis). Indeed, a positive relationship between Omin-C and the microbial biomass pool was found in 2015. However, this relationship was lost in 2016. So, why was this relationship not maintained? Although microbes are considered the main contributor to Omin-C, the size of the clay and silt fraction will eventually determine the extent of C that can be stored in this pool (Six and others 2002). The values that we obtained for C content in the clay/silt fraction in 2016 were similar to values that reflect C saturation in grasslands soils, when maximal stabilization is reached (Feng and others 2013). Indeed, in 2016, the C content showed a positive linear relationship with the clay/silt fraction for ambient and drought treatments with no significant difference between the slope of both treatments (not shown). However, this relationship was not observed in 2015, suggesting that saturation with C was not reached, but instead, that Omin-C was still influenced by microbial biomass. Our results further indicate that the Omin-C pool at this site was dynamic suggesting that formation of Omin-C can occur rather rapidly when the clay and silt fraction has yet to reach saturation.

We also observed that the increase in legume biomass between the first and second year was positively related to the increase in Omin-C (Figure 5) and more generally to the amount of Omin-C throughout the whole experiment (*R* ^*2*^ = 0.38, *P* < 0.001), whereas legume biomass was not affected by the drought treatment. We hypothesized that drought effects on Omin-C would be mediated by plant functional groups that vary in litter quality and rhizodeposition (third hypothesis). Possibly, litter and rhizodeposition of the legumes had lower C/N ratios, which may have stimulated microbial growth efficiency and stimulated the formation of Omin-C (Cotrufo and others 2013). This was confirmed in our experiment by the positive relationship between legume biomass and both Pom-N and MBN. The SEM further revealed that legumes contributed to the increase in Omin-C in the second year by directly increasing Pom-N and MBN. Legume biomass affected both Pom-N and MBN, whereas soil moisture was positively related to MBN, explaining 55% of the variation in Omin-C increase in the second year.

At our field site, legume biomass was relatively low compared to other functional groups and the increase in Omin-C observed in our experiment is therefore surprising for such a short period of time. However, previous experiments have shown a large increase in C with legumes presence, both in long- (Fornara and Tilman 2008) and short-term studies (De Deyn and others 2009), even when abundance of legumes is low (De Deyn and others 2011). By alleviating N limitation to plants and microbes, legumes could increase belowground C inputs and SOC formation (Fornara and Tilman 2008). Furthermore, our grassland site mostly consisted of cool season legumes that are more abundant during the winter and early spring, whereas our measurements of functional group biomass were done at peak summer time. Our results suggest that, besides the inherent protection offered by binding organic matter to soil minerals, plants can also affect the size of this C pool on a short time scale, making this pool more dynamic than previously thought.

## Conclusion

Our experiment showed that despite strong drought-induced reductions in aboveground biomass at our grassland site, soil C pools were not affected. This indicates limited drought legacy effects on soil C, although the large variability in soil moisture and other resources at our field site could have masked smaller drought effects. Microbes correlated with the size of Omin-C during the first year of the experiment, but this disappeared during the wetter second year, where Omin-C appeared to have reached saturation levels. Although drought reduced biomass of main functional groups, inputs into the soil were maintained most likely due to an increased allocation of C belowground. Legume biomass was associated with increased Omin-C, Pom-N and microbial N in the soil, possibly increasing microbial C use efficiency and causing greater allocation of C to organo-mineral complexes. Indeed our SEM revealed that legume biomass explained much of the large increase in Omin-C in the second year, through a greater supply of N-rich litter (greater Pom-N) and increasing MBN in soil. Overall, our experiment demonstrates the limited effect of drought on soil C pools but indicates a high importance of legumes and microbes for soil C formation in grasslands. The relative short time scale (two years) of our experiment might have been a reason behind the nonsignificant effect of drought on Omin-C. Longer experiments are needed to further verify the results we obtained. However, drought had no effect on the relatively faster turned-over C pool, Pom-C, further indicating the overall limited effect of drought on soil C cycling.

## Electronic supplementary material

Below is the link to the electronic supplementary material.

Supplementary material 1 (DOCX 184 kb)
