# Supplementary material for: Mineral-Associated Soil Carbon is Resistant to Drought but Sensitive to Legumes and Microbial Biomass in an Australian Grassland
Source: Ecosystems. 2017 Apr 25;21(2):349–59. doi: 10.1007/s10021-017-0152-x (PMC5840236; doi:10.1007/s10021-017-0152-x)
Supplement: Supplementary file 1 — Supplementary material 1 (DOCX 184 kb) [file 10021_2017_152_MOESM1_ESM.docx]

**Electronic Supporting information**

| **Table S1.** List of plant species identified during sampling in January 2015 and January 2016. Each species functional group and photosynthetic pathway is indicated. | | |
| --- | --- | --- |
| Species | Functional group | Photosynthetic pathway |
| *Chenopodium murale* | Forb | C_3_ |
| *Desmodium spp.* | Legume | C_3_ |
| *Hypochaeris radicata* L. | Forb | C_3_ |
| *Lepidium spp.* | Forb | C_3_ |
| *Medicago polymorpha* | Legume | C_3_ |
| *Medicago sativa* | Legume | C_3_ |
| *Modiola carolinensis* | Forb | C_3_ |
| *Bryophyta* | Forb | C_3_ |
| *Plantago lanceolata* | Forb | C_3_ |
| *Portulaca oleracea L*. | Forb | C_3_ |
| *Rumex brownii* | Forb | C_3_ |
| *Senecio madagascariensis* | Forb | C_3_ |
| *Trifolium spp.* | Legume | C_3_ |
| *Bromus spp.* | Grass | C_3_ |
| *Ehrharta erecta* | Grass | C_3_ |
| *Lolium rigidum* | Grass | C_3_ |
| *Microlaena stipoides* | Grass | C_3_ |
| *Bothriochloa macra* | Grass | C_4_ |
| *Chloris gayana* | Grass | C_4_ |
| *Cynodon dactylon* | Grass | C_4_ |
| *Cyperus brevifolius* | Grass | C_4_ |
| *Eragrostis brownii* | Grass | C_4_ |
| *Paspalum spp.* | Grass | C_4_ |
| *Setaria incrassata* | Grass | C_4_ |
| *Sporobolus africanus* | Grass | C_4_ |
| *Themeda triandra* | Grass | C_4_ |

Figure S1. Structural equation model containing all a priori hypothetical pathways. Abbreviations: MBC (microbial biomass carbon); MBN (microbial biomass nitrogen); Omin-C (organo-mineral carbon); Pom-C (particulate organic carbon); Pom-N (particulate organic nitrogen). The numbers in the arrows denote references used to support our predictions (*see* below). Boxes represent changes in pools between 2015 and 2016.


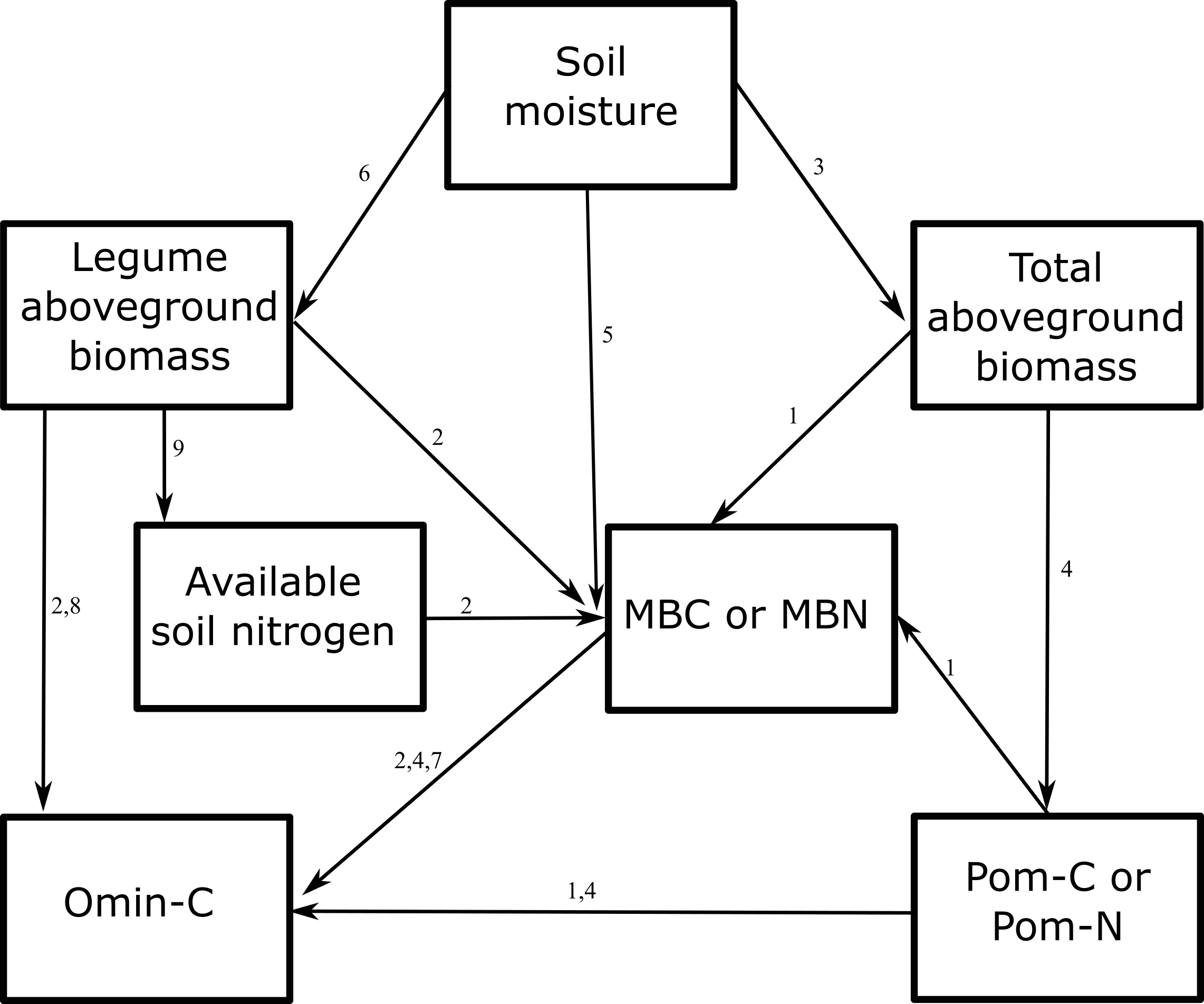


**References:**

1. Cotrufo, M. F., J. L. Soong, A. J. Horton, E. E. Campbell, M. L. Haddix, D. H. Wall and W. J. Parton (2015). "Formation of soil organic matter via biochemical and physical pathways of litter mass loss." Nature Geoscience **8**(10): 776-779.
2. Cotrufo, M. F., M. D. Wallenstein, C. M. Boot, K. Denef and E. Paul (2013). "The Microbial Efficiency-Matrix Stabilization (MEMS) framework integrates plant litter decomposition with soil organic matter stabilization: do labile plant inputs form stable soil organic matter?" Global Change Biology **19**(4): 988-995.
3. Hoover, D. L. and B. M. Rogers (2016). "Not all droughts are created equal: the impacts of interannual drought pattern and magnitude on grassland carbon cycling." Global Change Biology **22**(5): 1809-1820.
4. Kögel-Knabner, I. (2017). "The macromolecular organic composition of plant and microbial residues as inputs to soil organic matter: Fourteen years on." Soil Biology and Biochemistry **105**: A3-A8.
5. Schimel, J., T. C. Balser and M. Wallenstein (2007). "Microbial stress-response physiology and its implications for ecosystem function." Ecology **88**(6): 1386-1394.
6. Signarbieux, C. and U. Feller (2012). "Effects of an extended drought period on physiological properties of grassland species in the field." Journal of Plant Research **125**(2): 251-261.
7. Solomon, D., J. Lehmann, J. Harden, J. Wang, J. Kinyangi, K. Heymann, C. Karunakaran, Y. Lu, S. Wirick and C. Jacobsen (2012). "Micro- and nano-environments of carbon sequestration: Multi-element STXM–NEXAFS spectromicroscopy assessment of microbial carbon and mineral associations." Chemical Geology **329**(0): 53-73.
8. De Deyn, G. B., R. S. Shiel, N. J. Ostle, N. P. McNamara, S. Oakley, I. Young, C. Freeman, N. Fenner, H. Quirk and R. D. Bardgett (2011). "Additional carbon sequestration benefits of grassland diversity restoration." Journal of Applied Ecology **48**(3): 600-608.
9. Creme, A., A. Chabbi, F. Gastal and C. Rumpel (2016). "Biogeochemical nature of grassland soil organic matter under plant communities with two nitrogen sources." Plant and Soil: 1-13.
